# Impact of SARS-CoV-2 mRNA vaccine on arthritis condition in rheumatoid arthritis

**DOI:** 10.3389/fimmu.2023.1256655

**Published:** 2023-08-25

**Authors:** Ayuko Takatani, Naoki Iwamoto, Serina Koto, Toshiyuki Aramaki, Kaoru Terada, Yukitaka Ueki, Atsushi Kawakami, Katsumi Eguchi

**Affiliations:** ^1^ Rheumatic Disease Center, Sasebo Chuo Hospital, Sasebo, Japan; ^2^ Department of Immunology and Rheumatology, Division of Advanced Preventive Medical Sciences, Nagasaki University Graduate School of Biomedical Sciences, Nagasaki, Japan

**Keywords:** rheumatoid arthritis, SARS-CoV-2 mRNA vaccine, mRNA vaccine, arthralgia, COVID-19

## Abstract

**Background:**

The SARS-CoV-2 mRNA vaccine has been reported to cause various adverse reactions, including the development or exacerbation of autoimmune diseases, but the adverse reactions and the effects of the vaccines on disease activity in patients with rheumatoid arthritis (RA) remain unknown. We therefore investigated the arthritis condition in RA patients after SARS-CoV-2 vaccination.

**Methods:**

RA patients who visited our hospital from January to April 2022 completed a questionnaire regarding adverse reactions to the SARS-CoV-2 vaccine. We compared the frequency and duration of post-vaccination arthralgia between RA patients and health care workers in our hospital. For the RA patients who reported post-vaccination arthralgia, we collected medical records for the 6 months after vaccination.

**Results:**

Of the 1198 vaccinated RA patients, 256 (21.4%) had systemic inflammatory symptoms, 18 (1.5%) had allergies including urticaria and asthma, and 37 (3.1%) had arthralgia. A few patients had extra-articular manifestations such as acute exacerbation of interstitial lung disease. Compared with health care workers, RA patients more frequently developed arthralgia, and the arthralgia was longer lasting than that in controls: only 9 (0.8%) of the 1117 health care workers reported arthralgia, and all cases resolved within 3 days. Data from 31 of the 37 RA patients with post-vaccination arthralgia were further analyzed; in these patients, disease activity was highest after 2 months, and 10 patients required additional DMARDs within 6 months. The proportion of concomitant use of PSL at vaccination was higher in these patients. No patients on biological DMARDs or targeted synthetic DMARDs prior to vaccination needed additional DMARDs or a change of regimen.

**Conclusion:**

RA patients had more frequent and longer-lasting arthralgia after vaccination than healthy subjects, and one-third of patients with post-vaccination arthralgia required additional DMARDs. Although the SARS-CoV-2 mRNA vaccine was administered safely in most RA patients, in some patients RA symptoms may worsen after vaccination.

## Introduction

Coronavirus Disease 2019 (COVID-19) is an infectious disease caused by severe acute respiratory syndrome coronavirus 2 (SARS-CoV-2), a novel coronavirus discovered in Wuhan, China in December 2019 (1). COVID-19 causes flu-like symptoms such as fever and pharyngitis, but it can be severe, especially in the elderly or in those with underlying diseases such as diabetes, and can lead to acute respiratory distress syndrome, multiple organ failure and even death (2).

The COVID-19 pandemic led to the quick development and approval of SARS-CoV-2 mRNA vaccines. Although the vaccines are expected to prevent the onset and mitigate the severity of COVID-19 (3), adverse reactions have been reported, including the development and exacerbation of immune-related diseases (4). SARS-CoV-2 mRNA vaccine induces IFN-γ production via innate immune system (5, 6). Although the detailed mechanism by which vaccines cause immune-related diseases is not elucidated, abnormally high production of IFN-γ can trigger cytokine storm syndrome and autoinflammatory/autoimmune diseases (7, 8).

Rheumatoid arthritis (RA) is an autoimmune disease affecting the joints, estimated to affects approximately 0.5% to 1% of the global population. Given the large number of individuals affected by RA, it is crucial to understand the characteristics of adverse events of SARS-CoV-2 mRNA vaccine and their effects on disease activity in RA patients. Vaccines often cause arthralgia as an adverse event, but so far few data on the clinical course of arthralgia after vaccination in RA patients have been available.

Therefore, we investigated the adverse events and the course of arthralgia after SARS-CoV-2 mRNA vaccination in RA patients in this study. This study aimed to clarify the characteristics of adverse events by SARS-CoV-2 vaccine and the impact on disease activity in patients with RA.

## Patients and methods

### Patients

We administered a questionnaire about vaccination to RA patients who visited Sasebo Chuo Hospital from 11 January 2022 to 22 April 2022. A total of 1265 RA patients responded to our questionnaire and were enrolled in this study. All patients had a current diagnosis of RA based on the 2010 American College of Rheumatology (ACR)/European League against Rheumatism (EULAR) classification criteria for RA (9). We also analyzed the frequency of adverse events, including the appearance or worsening of arthralgia, among health care workers who were working in our hospital using a questionnaire previously administered from 19 April 2021 to 7 May 2021.

We collected patient data, including the date and type of vaccination, the disease duration of RA, positivity of rheumatoid factor (RF) and anti-citrullinated protein antibodies (ACPA), and the use of disease-modifying antirheumatic drugs (DMARDs) at the time of vaccination in RA patients who reported having post-vaccination arthralgia in the questionnaire. This study was approved by the ethics committee of Sasebo Chuo Hospital (approved number: 2021-45, 2023-19).

### Questionnaire

To evaluate the adverse symptoms after vaccination, a comprehensive questionnaire including information about the vaccine and subsequent symptoms was developed. The questionnaire included items about the vaccination status and number of doses of respondents, and symptoms such as fever, headache, allergy, and the appearance or worsening of arthralgia that had appeared within 2 weeks after vaccination (**Supplementary Data 1**).

### Clinical assessment

In RA patients who reported the appearance or worsening of arthralgia after vaccination, the patients’ clinical disease activity was assessed using the Disease Activity Score in 28 joints-erythrocyte sedimentation rate (DAS28-ESR) and the Clinical Disease Activity Index (CDAI) from the pre-vaccination to the 6-month post-vaccination visit.

### Statistical analysis

GraphPad prism software (GraphPad Software, San Diego, CA) was used for the statistical analyses. Normal distribution of the data was confirmed using the Kolmogorov-Smirnov test. Continuous variables were compared by Student’s paired t-test or Wilcoxon matched-pairs signed rank test (non-parametric), and the categorical variables were compared by Fisher’s exact test. All data are expressed as the medians and interquartile ranges or the numbers and percentages. A P values<0.05 were considered statistically significant.

## Results

### Characteristics of adverse events of SARS-CoV-2 mRNA vaccine

The demographic characteristics and adverse events of RA patients and health care workers are summarized in **Table 1**. A total of 1265 RA patients (994 females) responded to the questionnaire. Of these, 1190 patients (94.1%) were fully vaccinated (two doses), 8 patients (0.6%) were half vaccinated (only one dose) and 67 patients (5.3%) were unvaccinated. Of the 1198 RA patients who received at least one dose of the vaccine, 312 patients (26.0%) reported some adverse events after the first and/or second dose. The adverse events included systemic inflammatory symptoms such as fever, headache, and fatigue in 256 cases (21.4%), allergic symptoms such as urticaria and bronchial asthma in 18 cases (1.5%), and arthralgia in 37 cases (3.1%).

**Table 1:** Demographic characteristics and adverse events of vaccinated RA patients and health care workers.

	vaccinated RA patients (n=1198)	vaccinated health care workers (n=1117)	*p*-value
age, years, median (IQR)	67 (58-74)	38 (28-47)	<0.0001 *
female, n (%)	939 (78.4)	802 (71.8)	0.0003 *
partially vaccinated (only 1 time), n (%)	8 (0.7)	13 (1.2)	–
fully vaccinated (2 times), n (%)	1190 (99.3)	1104 (98.8)	0.27
some adverse events, n (%)	312 (26.0)	876 (77.0)	<0.0001 *
systemic inflammation symptoms (fever, headache, fatigue, et al), n (%)	256 (21.4)	841 (74.0)	<0.0001 *
allergy (urticaria, asthma, et al), n (%)	18 (1.5)	20 (1.8)	0.63
axillary lymph node swelling and/or tenderness, n (%)	11 (0.9)	12 (1.1)	0.84
arthralgia, n (%)	37 (3.1)	9 (0.8)	<0.0001 *
other symptoms, n (%)	31 (2.6)	–	–
nausea/vomiting, n (%)	6 (0.5)	–	–
anorexia, n (%)	4 (0.3)	–	–
paresthesia of limbs, n (%)	4 (0.3)	–	–
dizziness/vertigo, n (%)	3 (0.3)	–	–
acute exacerbation of ILD, n (%)	2 (0.2)	–	–
drowsiness, n (%)	2 (0.2)	–	–
rheumatoid nodule, n (%)	1 (0.1)	–	–
acute pleural effusion, n (%)	1 (0.1)	–	–
dyssomnia, (%)	1 (0.1)	–	–
tinnitus, n (%)	1 (0.1)	–	–
paranasal sinusitis, n (%)	1 (0.1)	–	–
palpitation, n (%)	1 (0.1)	–	–
diarrhea, n (%)	1 (0.1)	–	–
abdominal pain, n (%)	1 (0.1)	–	–
hematuria, n (%)	1 (0.1)	–	–
abnormal uterine bleeding, n (%)	1 (0.1)	–	–

IQR, interquartile range; RA, rheumatoid arthritis; ILD, interstitial lung disease *P<0.05.

### Comparison of adverse events including features of arthralgia after SARS-CoV-2 mRNA vaccination between RA patients and health care workers

We next compared the frequency and duration of arthralgia following vaccination between patients and healthy individuals (**Table 1**). As healthy controls, 1117 of the health care workers including nurses and doctors who worked in our hospital were included in this study. Their median age of them was 38 years old (IQR 28–47), 802 (71.8%) were female, and 1104 (98.8%) received with two doses of the vaccine. Adverse events included systemic inflammatory symptoms in 841 cases (74.0%), allergic symptoms in 20 cases (1.8%), and arthralgia in 9 cases (0.8%). As compared with RA patients, more of the health care workers reported systemic inflammatory symptoms (74.0% vs 21.4%, respectively, p<0.0001), and fewer reported arthralgia (0.8% vs 3.1%, respectively, p<0.0001). Moreover, arthralgia in the RA patient group improved within 3 days in only 4 patients and persisted for more than one month in 9 patients, while arthralgia in the health care worker group disappeared within 3 days in all cases (**Table 2**).

**Table 2:** Frequency and duration of arthralgia after receiving SARS-CoV-2 mRNA vaccine.

	vaccinated RA patients (n=1198)	vaccinated health care workers (n=1117)	*p*-value
arthralgia, n (%)	37 (3.1)	9 (0.8)	<0.0001 *
≦ 3 days, n	4	9	<0.0001 *
4 days - 1 week, n	1	0	>0.99
≧ 1 month, n	9	0	0.17

*P<0.05.

### Clinical course of RA patients with the appearance or worsening of arthralgia caused by vaccination

**Figure 1:**
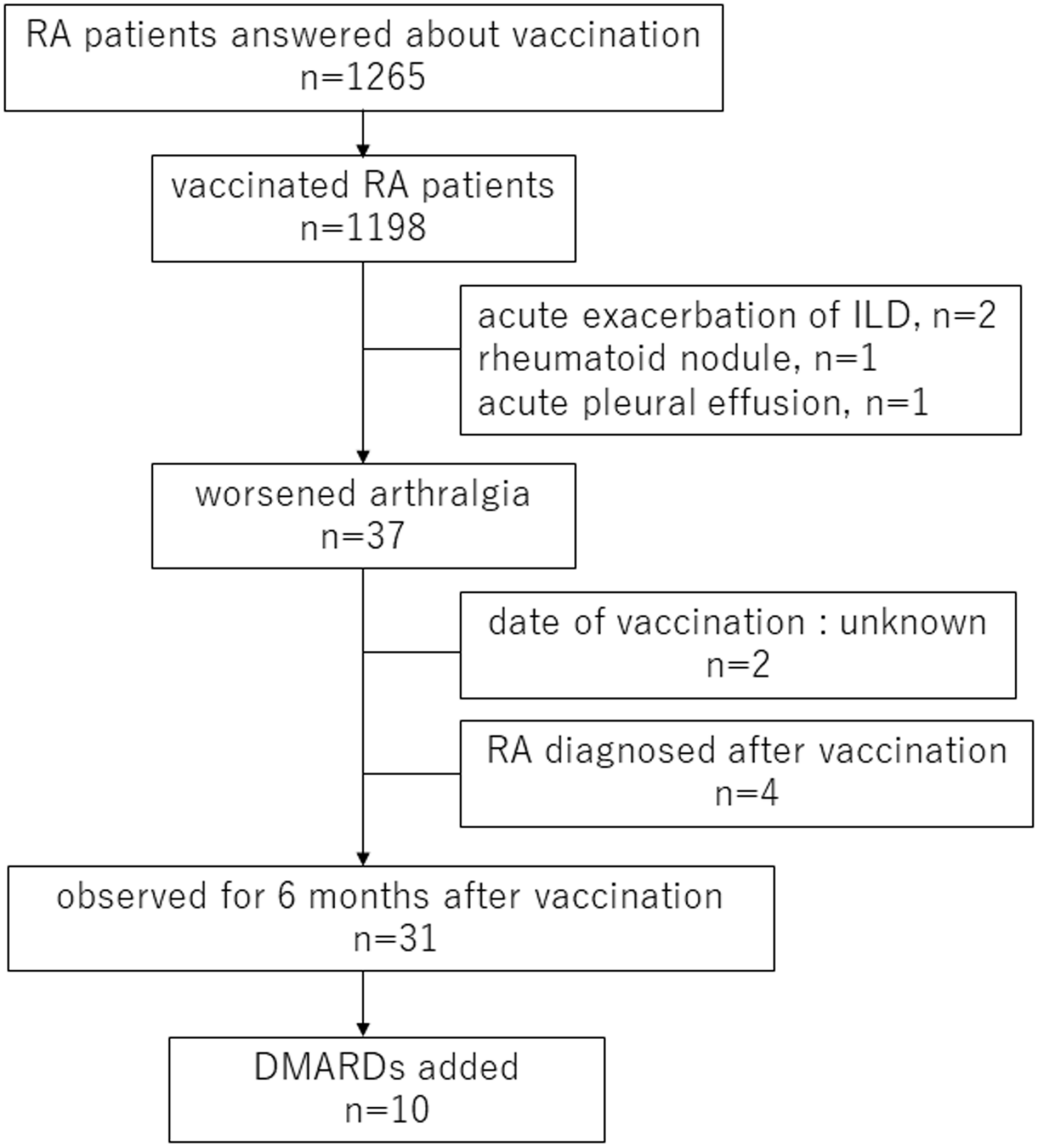
Flow chart of RA patients in this study RA, rheumatoid arthritis; ILD, interstitial lung disease; DMARDs, disease-modifying antirheumatic drugs.

**Figure 1** shows the study flowchart and clinical course of RA patients who showed disease exacerbation including RA-related organ manifestations such as interstitial lung disease (ILD). Regarding extra-articular manifestations, 2 cases of acute exacerbation of ILD, 1 case of subcutaneous rheumatoid nodule and 1 case of acute pleural effusion after vaccination were identified. Thirty-seven RA patients showed the appearance or worsening of arthralgia after vaccination. Of these patients, 4 individuals were newly diagnosed with RA after vaccination, 2 of whom had no prior experience of arthralgia.

Our present analysis specifically focused on the RA patients who were already diagnosed with RA at the vaccination. Thus, among the 37 RA patients who experienced arthralgia, 4 patients newly diagnosed as RA after vaccination and the 2 patients lack of the vaccination date were excluded and the clinical course of these 31 RA patients was reviewed from the pre-vaccination to the 6-month post-vaccination visit. Their median age of them was 64 years old (IQR 51.5–70), 21 (67.7%) were female, and 31 (100%) received with two doses of vaccination. Twenty-five patients were RF-positive (80.6%), and 19 patients were ACPA-positive (61.3%). Systemic inflammatory symptoms were observed after vaccination in 11 patients (29.7%) and allergic symptoms were observed in 1 patient (2.7%). Arthralgia worsened only after the first vaccination in 2 patients, only after the second vaccination in 13 patients, and after both vaccinations in 10 patients; the relation between arthralgia and the two vaccinations was unknown in 6 patients. Twenty-three patients received the Pfizer-BioTech COVID-19 vaccine (BNT162b2), 1 patient received the Moderna COVID-19 vaccine (mRNA-1273), and 11 patients were not sure which vaccine they received. Pre-vaccination treatment included 8 cases of biological DMARDs (bDMARDs) or targeted synthetic DMARDs (tsDMARDs), 21 cases of MTX and 6 cases of other conventional synthetic DMARDs (csDMARDs). Seven patients were concomitantly using PSL (**Table 3**).

**Table 3:** Demographic characteristics of RA patients observed for 6 months after vaccination.

	RA patients observed for 6 months after vaccination (n=31)	DMARDs added group (n=10)	no DMARDs added group (n=21)	*p*-value
age, years, median (IQR)	64 (51.5-70)	58 (51.3-68.8)	67 (53.0-72.0)	0.47
female, n (%)	21 (67.7)	4 (40.0)	6 (28.6)	0.13
duration of RA, year, median (IQR)	10 (5-16)	11 (5.8-17.5)	10 (4-10)	0.28
RF positivity, n (%)	25 (80.6)	8 (80.0)	17 (81.0)	>0.99
titer, IU/ml, median (IQR)	63.8 (26.2-126.6)	51.1 (30.2-123.9)	68.1 (26.7-117.2)	0.78
anti-CCP antibody positivity, n (%)	19 (61.3)	6 (60.0)	13 (61.9)	>0.99
titer, U/ml, median (IQR)	21 (1.2-150)	76 (1.6-143)	16.5 (1.08-150.5)	0.97
fully vaccinated (2 times), n (%)	31 (100)	10 (100)	21 (100)	–
systemic inflammation symptoms, n (%)	11 (29.7)	3 (30.0)	8 (38.1)	>0.99
allergy, n (%)	1 (2.7)	0 (0)	1 (4.8)	>0.99
timing of arthralgia after vaccination				
only after 1st vaccination, n (%)	2 (6.5)	0 (0)	2 (9.5)	>0.99
only after 2nd vaccination, n (%)	13 (41.9)	5 (50.0)	8 (38.1)	0.70
both after 1st and 2nd time, n (%)	10 (32.3)	5 (50.0)	5 (23.8)	0.22
unknown, n (%)	6 (19.4)	0 (0)	6 (28.6)	–
vaccine type				
Pfizer-BioTech vaccine, n (%)	20 (64.5)	6 (60.0)	14 (66.7)	>0.99
Moderna vaccine, n (%)	1 (3.2)	1 (10.0)	0 (0)	0.32
unknown, n (%)	10 (32.3)	2 (20.0)	8 (38.1)	–
disease activity score before vaccination				
DAS28-ESR, median (IQR)	2.64 (2.3-3.65)	2.8 (2.22-3.54)	2.55 (2.37-3.65)	0.98
CDAI, median (IQR)	4 (1-9.5)	3 (1.25-6.725)	4.5 (1-12)	0.55
medication before vaccination				
b/tsDMARDs, n (%)	8 (25.8)	0 (0)	8 (38.1)	0.03*
MTX, n (%)	21(67.7)	7 (70.0)	14 (66.7)	>0.99
other csDMARDs, n (%)	10 (32.3)	4 (40.0)	6 (28.6)	0.69
PSL combined with DMARDs, n (%)	7 (22.6)	5 (50.0)	2 (9.5)	<0.05

IQR, interquartile range; RA, rheumatoid arthritis; RF, rheumatoid factor; DMARDs, disease-modifying antirheumatic drugs; bDMARDs, biologic DMARDs; tsDMARDs, targeted synthetic DMARDs; csDMARDs, conventional synthetic DMARDs; MTX, methotrexate; PSL, prednisolone *P<0.05.

**Figure 2:**
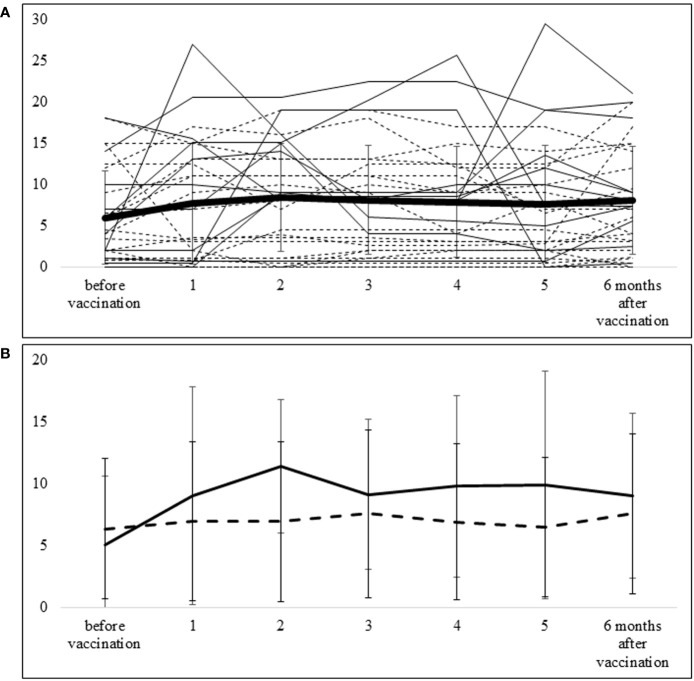
Course of CDAI from pre-vaccination to 6 months after vaccination **(A)** The thick line represents the mean ± SD of CDAI for all patients. The solid lines show CDAI values of each patient in the group requiring additional DMARDs, and the dotted lines show CDAI values of each patient in the group not requiring additional DMARDs. **(B)** The solid line shows the mean ± SD of CDAI in the group requiring additional DMARDs, and the dotted line shows the mean ± SD of CDAI in the group that did not require additional DMARDs.

Disease activity was elevated in some patients; the mean value was highest at 2 months after vaccination but was not significantly changed from pre-vaccination levels at the 6-month follow-up (**Figure 2A**). The median (IQR) DAS28-ESR score and CDAI were 2.64 (2.3–3.65) and 4.0 (1.0–9.5) at the time of vaccination, 3.34 (2.43–4.09) and 8.5 (2.0–13.5) at 2 months post-vaccination, and 3.15 (2.36–4.08) and 7.0 (2.75–13) at 6 months post-vaccination, respectively. No patients contracted COVID-19 within the 6-month follow-up period after vaccination.

We also conducted a comparison of the clinical characteristics between RA patients who experienced arthralgia after vaccination and RA patients without worsening arthralgia, for whom clinical characteristics data was available (n=971). The results revealed no significant difference in clinical characteristics between these two groups of RA patients (**Supplementary Table 1**).

### Factors associated with the requirement of additional DMARDs in patients with vaccination-induced arthralgia

Ten patients required additional or increased doses of DMARDs within 6 months after vaccination. Three patients required additional biologics (2 abatacept, 1 infliximab-BS), 6 patients received an increased dose of MTX, and 1 patient had additional iguratimod. The course of CDAI in patients with additional or increased doses of DMARDs (n=10) and those without additional DMARDs (n=21) is shown in **Figure 2B**. In the group without additional DMARDs, the mean (SD) CDAI change from baseline to 2 months was 0.59 (4.92), whereas in the additional DMARDs group, the corresponding value was 6.39 (8.21). With the additional treatment, this difference became smaller—i.e., the mean CDAI change from baseline to 6 months was 1.22 (4.67) in the group without additional DMARDs and 3.97 (6.67) in the group with additional DMARDs.

To investigate the factors associated with persistent worsening arthralgia, we compared the baseline characteristics between RA patients whose treatment remained unchanged during 6 months after vaccination and RA patients whose treatment needed to be changed specifically to suppress arthralgia (**Table 3**). The majority of variables were comparable between the two groups; however, there was a higher prevalence of patients using PSL concurrently with other DMARDs in the group that required additional DMARDs. Furthermore, none of the patients who were already on b/tsDMARDs treatment prior to vaccination needed to add or switch to different DMARDs or b/tsDMARDs.

## Discussion

In this study, we first investigated the adverse events of SARS-CoV-2 mRNA vaccine. The RA patient group had fewer systemic inflammatory symptoms but more arthralgia than the health care worker group. The lower occurrence of systemic inflammatory symptoms in the RA group might derive from the difference of age at the vaccination, because systemic inflammatory symptoms after vaccination are more common among younger people (10, 11). Additionally, medication such as NSAIDs, prednisolone, and DMARDs may have masked systemic inflammatory symptoms in RA patients. However, it is surprising that arthralgia was high prevalence even under these medications.

The prevalence of arthralgia after receiving the SARS-CoV-2 vaccine has been reported to be 0.83–46.4% in the general population (12–16). In a small number of studies, it has been reported that arthralgia is a more frequent response to vaccination in patients with autoimmune diseases, including RA, compared with healthy controls. Furer et al. reported that the prevalence of arthralgia after the first vaccination was 3.42% in the autoimmune disease group and 0.83% in the control group; after the second vaccination, those percentages were 7.32% and 4.96%, respectively (16). Lars Erik Bartels et al. reported that the prevalence of post-vaccination arthralgia was higher in an autoimmune disease group consisting of patients with RA and SLE than in healthy controls, with an odds ratio of 2.3 (95% CI 1.7–3.0) (17). In our present study, we also observed that RA patients experienced a higher incidence of arthralgia compared to the health care worker group, supporting the finding that arthralgia from the SARS-CoV-2 vaccine is more likely to occur in RA patients. The reason for the high frequency of post-vaccination arthralgia in RA patients is unclear. In RA patients, if arthritis remains, they may be more likely to experience arthralgia due to immune-activated conditions such as vaccinations or infections, or they may be more likely to feel arthralgia because they are more concerned about arthralgia than healthy individuals.

Interestingly, not only the frequency but also the duration of arthralgia differed between the two groups. Arthralgia disappeared within 3 days in all health care workers who reported it, whereas arthralgia in RA patients often persisted for longer days. It is controversial whether the SARS-CoV-2 vaccine exacerbates autoimmune diseases including RA (4, 18). While our study also cannot definitively establish whether the SARS-CoV-2 vaccine directly worsened RA, it is possible that mRNA vaccination could serve as an aggravating factor in RA as, for instance, smoking, obesity and environmental factors might (19). Recent study has shown the distinct different metabolic alterations of local lymph node stromal microenvironment in RA patients compared with healthy controls (20). Thus, the present result suggests that for some RA patients, the SARS-CoV-2 vaccine might act as a potential trigger for exacerbation of arthritis compared with healthy individuals. To ensure the safe use of mRNA vaccinations, including the SARS-CoV-2 vaccine, in RA patients, larger-scale studies are needed in the future. Interestingly, none of the patients who were already using b/tsDMARDs before vaccination required additional DMARDs treatment, suggesting that stronger immunosuppression might help prevent vaccine-induced exacerbation of RA.

The limitations of this study were the short observation period and the possibility of recall bias, as the RA patients and the health care worker group were administered questionnaires at different periods. In addition, we did not analyze the comorbidities of autoimmune disease, such as Sjogren syndrome and systemic lupus erythematosus. These autoimmune conditions can also contribute to arthralgia and may potentially be influenced by vaccination. This is not only for RA patients, but also for health care workers. Furthermore, the clinical course of unvaccinated RA patients has not been studied, so we have not been able to confirm whether vaccinated RA patients experience more flare-ups.

Many of the patients who had worsening arthralgia and whose records for the following 6 months were collected were vaccinated with Pfizer’s vaccine; relatively few received Moderna’s vaccine. Indeed, most people in Japan at that time were vaccinated with the Pfizer-BioTech vaccine, not the Moderna vaccine. Therefore, this study cannot discuss differences in adverse events due to vaccine type.

In conclusion, our study provides evidence that RA patients experience more frequent and longer-lasting arthralgia as a result of SARS-CoV-2 vaccination. Most of the RA patients were able to receive the vaccine safely. However approximately one-third of RA patients who had arthralgia after vaccination required additional DMARDs within the following 6 months. We should be prepared for this tendency for more prolonged and frequent arthralgia as compared with the general population; in some RA patients, prescription of additional DMARDs post-vaccination may be necessary to manage the increased disease activity. Further research and larger-scale studies are warranted to explore strategies for minimizing arthralgia and optimizing vaccination outcomes in RA patients.

## Data availability statement

The raw data supporting the conclusions of this article will be made available by the authors, without undue reservation.

## Ethics statement

The studies involving humans were approved by the ethics committee of Sasebo Chuo Hospital (approved number: 2021-45, 2023-19). The studies were conducted in accordance with the local legislation and institutional requirements. The participants provided their written informed consent to participate in this study.

## Author contributions

AT: Writing – original draft. NI: Writing – original draft. SK: Writing – review & editing. TA: Writing – review & editing. KT: Writing – review & editing. YU: Writing – review & editing, Supervision. AK: Writing – review & editing, Supervision. KE: Writing – review & editing, Conceptualization, Supervision.

## References

[1] Guan WJ Ni ZY Hu Y Liang WH Ou CQ He JX. Clinical characteristics of coronavirus disease 2019 in China. N Engl J Med (2020) 382(18):1708–20. doi: 10.1056/NEJMoa2002032 PMC7092819 32109013

[2] Sinha P Calfee CS Cherian S Brealey D Cutler S King C. Prevalence of phenotypes of acute respiratory distress syndrome in critically ill patients with COVID-19: a prospective observational study. Lancet Respir Med (2020) 8(12):1209–18. doi: 10.1016/S2213-2600(20)30366-0 PMC7718296 32861275

[3] Kuhlmann C Mayer CK Claassen M Maponga T Burgers WA Keeton R. Breakthrough infections with SARS-CoV-2 omicron despite mRNA vaccine booster dose. Lancet (2022) 399(10325):625–6. doi: 10.1016/S0140-6736(22)00090-3 PMC8765759 35063123

[4] Watad A De Marco G Mahajna H Druyan A Eltity M Hijazi N. Immune-mediated disease flares or new-onset disease in 27 subjects following mRNA/DNA SARS-CoV-2 vaccination. Vaccines (Basel) (2021) 9(5):435–58. doi: 10.3390/vaccines9050435 PMC8146571 33946748

[5] Arunachalam PS Scott MKD Hagan T Li C Feng Y Wimmers F. Systems vaccinology of the BNT162b2 mRNA vaccine in humans. Nature (2021) 596(7872):410–6. doi: 10.1038/s41586-021-03791-x PMC8761119 34252919

[6] Pardi N Hogan MJ Porter FW Weissman D. mRNA vaccines - a new era in vaccinology. Nat Rev Drug Discov (2018) 17(4):261–79. doi: 10.1038/nrd.2017.243 PMC5906799 29326426

[7] Ivashkiv LB Donlin LT. Regulation of type I interferon responses. Nat Rev Immunol (2014) 14(1):36–49. doi: 10.1038/nri3581 24362405 PMC4084561

[8] Bindoli S Giollo A Galozzi P Doria A Sfriso P. Hyperinflammation after anti-SARS-CoV-2 mRNA/DNA vaccines successfully treated with anakinra: Case series and literature review. Exp Biol Med (Maywood) (2022) 247(4):338–44. doi: 10.1177/15353702211070290 PMC8899337 35068221

[9] Aletaha D Neogi T Silman AJ Funovits J Felson DT Bingham CO 3rd. 2010 rheumatoid arthritis classification criteria: an American College of Rheumatology/European League Against Rheumatism collaborative initiative. Ann Rheum Dis (2010) 69(9):1580–8. doi: 10.1136/ard.2010.138461 20699241

[10] Menni C Klaser K May A Polidori L Capdevila J Louca P. Vaccine side-effects and SARS-CoV-2 infection after vaccination in users of the COVID Symptom Study app in the UK: a prospective observational study. Lancet Infect Dis (2021) 21(7):939–49. doi: 10.1016/S1473-3099(21)00224-3 PMC8078878 33930320

[11] Frenck RW Jr. Klein NP Kitchin N Gurtman A Absalon J Lockhart S. Safety, immunogenicity, and efficacy of the BNT162b2 covid-19 vaccine in adolescents. N Engl J Med (2021) 385(3):239–50. doi: 10.1056/NEJMoa2107456 PMC8174030 34043894

[12] Kadali RAK Janagama R Peruru S Malayala SV. Side effects of BNT162b2 mRNA COVID-19 vaccine: A randomized, cross-sectional study with detailed self-reported symptoms from healthcare workers. Int J Infect Dis (2021) 106:376–81. doi: 10.1016/j.ijid.2021.04.047 PMC8049195 33866000

[13] Meo SA Bukhari IA Akram J Meo AS Klonoff DC. COVID-19 vaccines: comparison of biological, pharmacological characteristics and adverse effects of Pfizer/BioNTech and Moderna Vaccines. Eur Rev Med Pharmacol Sci (2021) 25(3):1663–9. doi: 10.26355/eurrev_202102_24877 33629336

[14] Amodio E Minutolo G Casuccio A Costantino C Graziano G Mazzucco W. Adverse reactions to anti-SARS-CoV-2 vaccine: A prospective cohort study based on an active surveillance system. Vaccines (Basel) (2022) 10(3):345–57. doi: 10.3390/vaccines10030345 PMC8954936 35334977

[15] Baydar O Ozen S Ozturk Sahin B Kokturk N Kitapci MT. Safety of an inactivated SARS-CoV-2 vaccine among healthcare workers in Turkey: an online survey. Balkan Med J (2022) 39(3):193–8. doi: 10.4274/balkanmedj.galenos.2022.2021-11-25 PMC9136550 35380034

[16] Furer V Eviatar T Zisman D Peleg H Paran D Levartovsky D. Immunogenicity and safety of the BNT162b2 mRNA COVID-19 vaccine in adult patients with autoimmune inflammatory rheumatic diseases and in the general population: a multicentre study. Ann Rheum Dis (2021) 80(10):1330–8. doi: 10.1136/annrheumdis-2021-220647 34127481

[17] Bartels LE Ammitzboll C Andersen JB Vils SR Mistegaard CE Johannsen AD. Local and systemic reactogenicity of COVID-19 vaccine BNT162b2 in patients with systemic lupus erythematosus and rheumatoid arthritis. Rheumatol Int (2021) 41(11):1925–31. doi: 10.1007/s00296-021-04972-7 PMC8412379 34476603

[18] Nakafero G Grainge MJ Card T Mallen CD Nguyen Van-Tam JS Williams HC. Is vaccination against Covid-19 associated with autoimmune rheumatic disease flare? A self-controlled case series analysis. Rheumatol (Oxford) (2022) 62:1445–50. doi: 10.1093/rheumatology/keac484 PMC10070057 36048896

[19] Arleevskaya M Takha E Petrov S Kazarian G Renaudineau Y Brooks W. Interplay of environmental, individual and genetic factors in rheumatoid arthritis provocation. Int J Mol Sci (2022) 23(15):8140–65. doi: 10.3390/ijms23158140 PMC9329780 35897715

[20] de Jong TA Semmelink JF Denis SW Bolt JW Maas M van de Sande MGH. Lower metabolic potential and impaired metabolic flexibility in human lymph node stromal cells from patients with rheumatoid arthritis. Cells (2022) 12(1):1–14. doi: 10.3390/cells12010001 PMC9818527 36611795

