# Supplementary material for: Impact of SARS-CoV-2 mRNA vaccine on arthritis condition in rheumatoid arthritis
Source: Front Immunol. 2023 Aug 25;14:1256655. doi: 10.3389/fimmu.2023.1256655 (PMC10492583; doi:10.3389/fimmu.2023.1256655)
Supplement: Supplementary file 1 [file Table_1.docx]

**Suppl table 1. Demographic characteristics of RA patients with and without post-vaccination arthralgia**

|  | RA patients with post-vaccination arthralgia (n=31) | RA patients without post-vaccination arthralgia (n=971) | *p*-value |
| --- | --- | --- | --- |
|  |  |  |  |
| age, years, median (IQR) | 64 (51.5-70) | 67 (57.0-74.0) | 0.13 |
| female, n (%) | 21 (67.7) | 769 (79.2) | 0.18 |
| duration of RA, year, median (IQR) | 10 (5-16) | 11 (5.0-18.0) | 0.53 |
| RF positivity, n (%) | 25 (80.6) | 702 (73.5) | 0.41 |
| titer, IU/ml, median (IQR) | 63.8 (26.2-126.6) | 48.7 (12.5-142.2) | 0.46 |
| anti-CCP antibody positivity, n (%) | 19 (61.3) | 672 (71.9) | 0.33 |
| titer, U/ml, median (IQR) | 21 (1.2-150) | 54 (2.5-217) | 0.33 |
| fully vaccinated (2 times), n (%) | 31 (100) | 969 (99.8) | >0.99 |
| systemic inflammation symptoms, n (%) | 11 (29.7) | 203 (20.9) | 0.07 |
| allergy, n (%) | 1 (2.7) | 12 (1.2) | 0.34 |
| disease activity score before vaccination | | | |
| CRP, median (IQR) | 0.12 (0.037-0.452) | 0.1 (0.0-0.2) | 0.34 |
| ESR, median (IQR) | 21 (8-36.5) | 19 (10-38) | 0.86 |
| DAS28-ESR, median (IQR) | 2.64 (2.3-3.65) | 3.0 (2.22-3.8) | 0.51 |
| CDAI, median (IQR) | 4 (1-9.5) | 5 (2.0-10.0) | 0.34 |
| medication before vaccination | | | |
| b/tsDMARDs, n (%) | 8 (25.8) | 405 (41.7) | 0.09 |
| MTX, n (%) | 21(67.7) | 642 (66.1) | >0.99 |
| other csDMARDs, n (%) | 10 (32.3) | 298 (30.7) | 0.84 |
| PSL combined with DMARDs, n (%) | 7 (22.6) | 198 (20.4) | 0.8207 |

IQR, interquartile range; RA, rheumatoid arthritis; RF, rheumatoid factor; DMARDs, disease-modifying antirheumatic drugs; bDMARDs, biologic DMARDs; tsDMARDs, targeted synthetic DMARDs; csDMARDs, conventional synthetic DMARDs; MTX, methotrexate; PSL, prednisolone *P<0.05
